# Changes and clinical correlation of diffusion tensor imaging parameters of compressed spinal cord and nerve root in patients with cervical spondylosis

**DOI:** 10.1186/s12880-022-00835-0

**Published:** 2022-06-03

**Authors:** Shuo Liang, Feng Yang, Yang Zhang, Huiyu Zhao, Xinyue Wang

**Affiliations:** 1 Department of Radiology, Central Hospital Affiliated to Shenyang Medical College, Shenyang Liaoning, China; 2 Department of Spine Surgery, Central Hospital Affiliated to Shenyang Medical College, Shenyang Liaoning, China

**Keywords:** Apparent diffusion coefficient, Cervical spondylosis, Diffusion tensor imaging, Fractional anisotropy, Magnetic resonance imaging

## Abstract

**Background:**

Diffusion tensor imaging (DTI) was used to quantitatively study the characteristics of the related spinal cord and nerve root compression parameters in patients with cervical spondylosis (CS), and diffusion tensor tractography (DTT) was used to visualize the spinal cord and nerve root and analyze their relevance to clinical evaluation.

**Methods:**

A total of 67 patients with CS and 30 healthy volunteers received 3.0 T magnetic resonance imaging. Cervical DTI and DTT were performed in all the participants, where the b value of DTI was set at 800 s/mm^2^. Fractional anisotropy (FA) and apparent diffusion coefficient (ADC) values of the spinal cord and cervical nerve roots were measured by using DTI. Patients with CS were scored according to the modified Japanese Orthopedic Association (mJOA) score.

**Results:**

In all the participants, the spinal cord and cervical nerve roots were clearly visible by DTT. Compared to the healthy volunteers, the FA values were significantly decreased and ADC values were significantly increased in patients with CS. mJOA score was significantly correlated with the DTI index (ADC and FA) values. Receiver operator characteristic curve analysis revealed that FA and ADC could identify mild, moderate, and severe CS.

**Conclusions:**

DTI parameters of cervical spinal cord and nerve root compression are associated with the clinical evaluation of patients with CS and may be helpful in assessing the severity of CS.

## Background

Cervical spondylosis (CS) is a common clinical disorder showing degenerative changes in the cervical vertebrae. Patients generally present with pain and stiffness in the neck along with symptoms of radiating pain such as in the upper limbs. CS tends to occur between 40 and 60 years of age [1] and may lead to severe dysfunction requiring surgery [2]. For cervical spondylotic myelopathy, early patients are mostly treated with conservative treatment. When spinal cord compression persists, the spinal cord signal gradually changes from moderate to high T2WI signal, indicating that the spinal cord compression degeneration injury has reached a relatively serious degree, and the prognosis is poor even after surgical treatment. Therefore, early and accurate diagnosis of CS and evaluation of severity benefits the treatment and prognosis of CS.

Magnetic resonance imaging (MRI), computed tomography (CT) and X-ray are the most commonly used diagnostic methods of CS at present. X-ray can accurately determine the stenosis of vertebral space and abnormal curvature of cervical spine. CT has the characteristics of high resolution, which can accurately determine uncoupled vertebral joint osteoplasia and spinal canal stenosis. MRI has excellent soft tissue resolution and is considered the best imaging method for diagnosing CS [3, 4]; conventional MRI findings and clinical symptoms are sometimes inconsistent, making accurate assessment of the severity of the spinal cord and nerve root compression in a timely manner challenging [5, 6]. When objective imaging evaluation is lacking, the curative effect of CS relies on patient’s subjective experience and clinical observation.

Diffusion tensor imaging (DTI) technology uses multiple diffusion-sensitive gradients in different directions to quantify the anisotropy of diffusion of water molecules and accurately display the morphological characteristics of the nerve fiber bundles [7, 8]. Currently, it can clearly identify the white matter tracts in brain tissue and the changes in peripheral nerve fibers [9, 10]. Studies have confirmed that DTI can sensitively reflect the changes in the diffusion anisotropy of water molecules in the nerve fiber bundles, which may provide information on the subtle pathophysiological changes of the living nerve fiber bundles, such as the reduction of the number of motor neurons, nerve cell atrophy and demyelination and other changes; this proves that DTI can better describe the severity of spinal cord injury [11–13]. DTI parameters include fractional anisotropy (FA) and apparent diffusion coefficient (ADC).

This study aimed to measure the FA and ADC values of compressed spinal cord and nerve root by DTI, thereby analyze the correlation between the FA and ADC values and the clinical score; moreover, the clinical values of the DTI parameters for evaluating the severity of CS have been discussed.

## Methods

### Study design and participants

The study included 67 consecutive patients diagnosed with CS based on clinical signs, symptoms and conventional MRI, including spinal cord and/or nerve root compression, in our hospital from December 2020 to July 2021. The main clinical manifestations were neck pain, dizziness, shoulder pain, and upper limb numbness, persisting longer than 3 months. Most of the patients showed slow onset, progressive or sudden aggravation, with varying degrees of spinal cord and cervical nerve root compression. Patients with trauma, infection, neoplasm,or other etiologies were excluded. Patients with CS were scored according to the modified Japanese Orthopaedic Association (mJOA) score.

Thirty volunteers comprised the control group, all of whom had no symptoms of spinal cord or cervical nerve root compression, imaging changes, history of neurological or psychiatric diseases, history of trauma, or contraindications for MRI. They were further divided into three groups by age, namely ≤ 30 years, 31–50 years, and ≥ 51 years, with 10 participants in each group.

The study followed the Declaration of Helsinki was approved by the Ethics Committee of Central Hospital affiliated to Shenyang Medical College. All participants provided written informed consent.

### Image acquisition

Imaging was performed using SIEMENS Magnetom Spectra 3.0 T MAGNETIC resonance scanner (SIEMENS Magnetom Spectra; Siemens Healthcare, Erlangen, Germany). Routine cervical spine sequence was followed for all scans. All patients underwent sagittal T2 weighted imaging (WI), T1WI, and axial T2WI scans using fast spin echo (FSE). Scan parameters were as follows: for sagittal T2WI, repetition time/echo time (TR/TE) 3200/99 ms; T1WI, TR/TE 380/11.3 ms; slice thickness 3.0 mm, field of view (FOV) 240 × 240 mm, acquisition matrix 320 × 256; axial T2WI, TR/TE 3500/90 ms, slice thickness 3.0 mm, FOV 300 × 100 mm, and acquisition matrix 320 × 224.

Axial imaging of cervical spine was performed using RESOLVE DTI sequences based on multi shot EPI. The scanning level was C3–C7. The diffusion sensitive gradient was set in 10 directions, and the parameters were: TR/TE 6680/80 ms; FOV 320 × 240 mm; matrix 160 × 112; slice thickness 2.0 mm; gap 0 mm; slice number 40; phase encode direction A >> P; concatenations 1; diffusion weighting coefficient (b) value 0, 800 s/mm^2^; bandwidth 679 Hz/Px; 2 averages; and scanning time 9 min 43 s.

After the RF excitation and the application of the diffusion gradient, the readout phase of the RESOLVE sequence is divided into two parts, namely Imaging Echo and Navigator Echo, in which the Imaging Echo is acquired in a similar way to the readout of the single shot DTI, except that a bipolar gradient is applied to the readout gradient Gr to achieve the number and position of the K-space segments. The Navigator Echo is used to obtain the phase information of the corresponding segment, which can be used for non-linear phase correction between segments or to decide on resampling in the case of large phase differences.

### Image processing and measurements

**Fig. 1:**
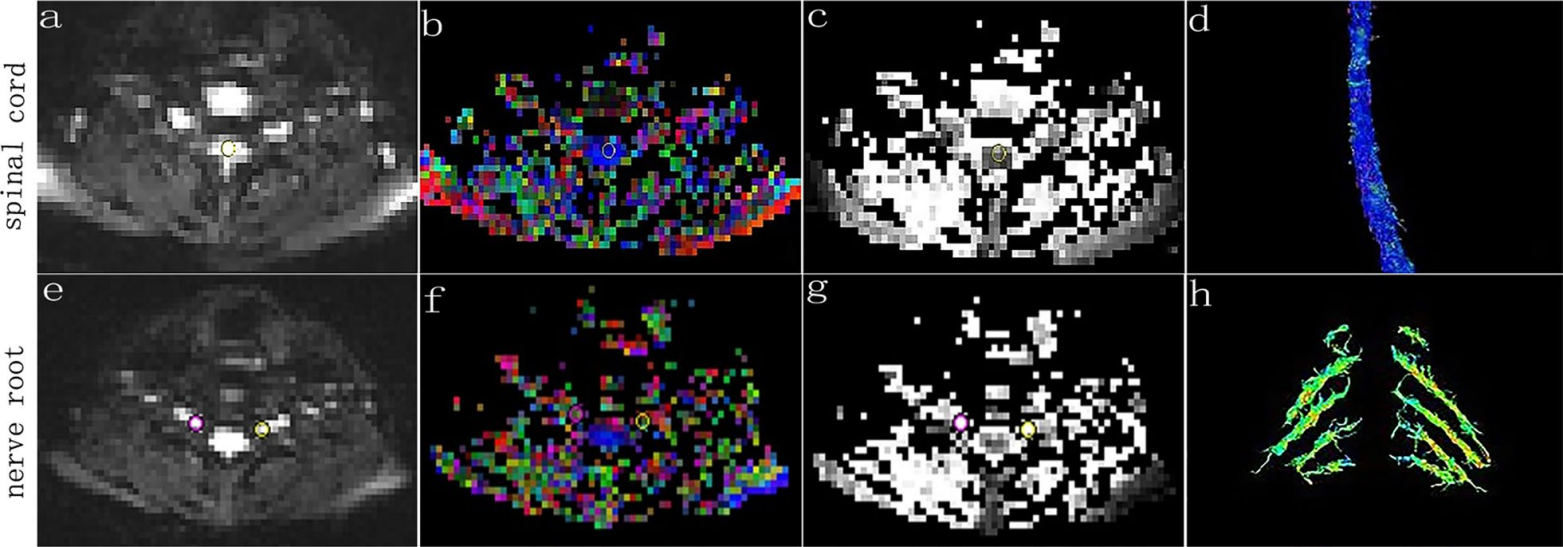
Measurement of diffusion tensor imaging parameters. **a**–**h** Spinal cord and nerve root images from a healthy volunteer. **a**, **e** Selected regions of interest of 2–4 pixels on the spinal cord and nerve root b0 images. **b**, **f** Fractional anisotropy images. **c**, **g** Apparent diffusion coefficient images. **d**, **h** Spinal cord and nerve root fiber tracts

All the data were stored on Syngo image workstation (Siemens Healthcare, Erlangen, Germany), and FA and ADC images were automatically generated. In the 3D Neuro mode of the DTI post-processing window, the DTI tensor images were transformed into the fusion mode, the seed points were drawn along the nerve fiber bundles in the sagittal position of the spinal cord and the coronal position of the nerve root. DTT images of the cervical spinal cord and the cervical nerve roots were generated by tractography. According to the degree of compression of the cervical spinal cord and cervical nerve root observed on conventional sagittal and axial T2WI images, two senior radiologists selected the most severe compression levels of cervical spinal cord and cervical nerve root, then the regions of interest (ROIs) were manually drawed in the central part of spinal cord and nerve root compression on FA and ADC maps, avoiding cerebrospinal fluid as much as possible. Each ROI was about 2–4 pixels in size (Fig. 1), FA and ADC values for each ROI were displayed automatically. FA and ADC values of spinal cord and nerve root (both sides) were measured at four levels from C3/4-C6/7 in healthy volunteers. FA and ADC values were measured blind by three radiologists, and the average value was taken as the final measurement result.

### Statistical analysis

All the statistical analyses were performed using SPSS Version 23.0 (Armonk, NY: IBM Corp.). The data are expressed as mean ± standard deviation. Normality of the data was tested and confirmed. One way analysis of variance (ANOVA) was used for multi-group comparisons. Paired-samples t-test was used for intra-group comparisons. Independent-samples t-test was used for comparison between the groups. Correlation was analyzed by using Pearson correlation analysis. Receiver operating characteristic (ROC) curve was drawn to calculate the area-under-the-curve (AUC) of CS of different severity, and the prediction threshold was determined. AUC > 0.5 has predictive value, and the higher the value, the better the ability to predict. AUC 0.5 to 0.7 was considered as having low predictive value, 0.7 to 0.9 medium predictive value, and above 0.9 high predictive value. *P* < 0.05 was considered statistically significant.

## Results

### Characteristics of the participants

The 67 patients with CS included 33 men and 34 women, with an average age of 47.1 years (range 25–67 years). According to the modified Japanese orthopedic association (mJOA) score [14], patients with CS were divided into 26 mild (mJOA ≥ 15), 29 moderate (mJOA = 12–14), and 12 severe (mJOA < 12) patients. The most severe spinal cord compression occurred at C3/4 level in six patients, C4/5 level in 15 patients, C5/6 level in 29 patients, and C6/7 level in 13 patients. Nerve root compression sites included both left (n = 22) and right (n = 33) sides, in the C5 (n = 10), C6 (n = 24), and C7 (n = 21) nerve roots. The average mJOA score was 13.13 ± 2.44. The control group included 13 men and 17 women with a mean age of 40.9 years (range 24–66 years).

**Fig. 2:**
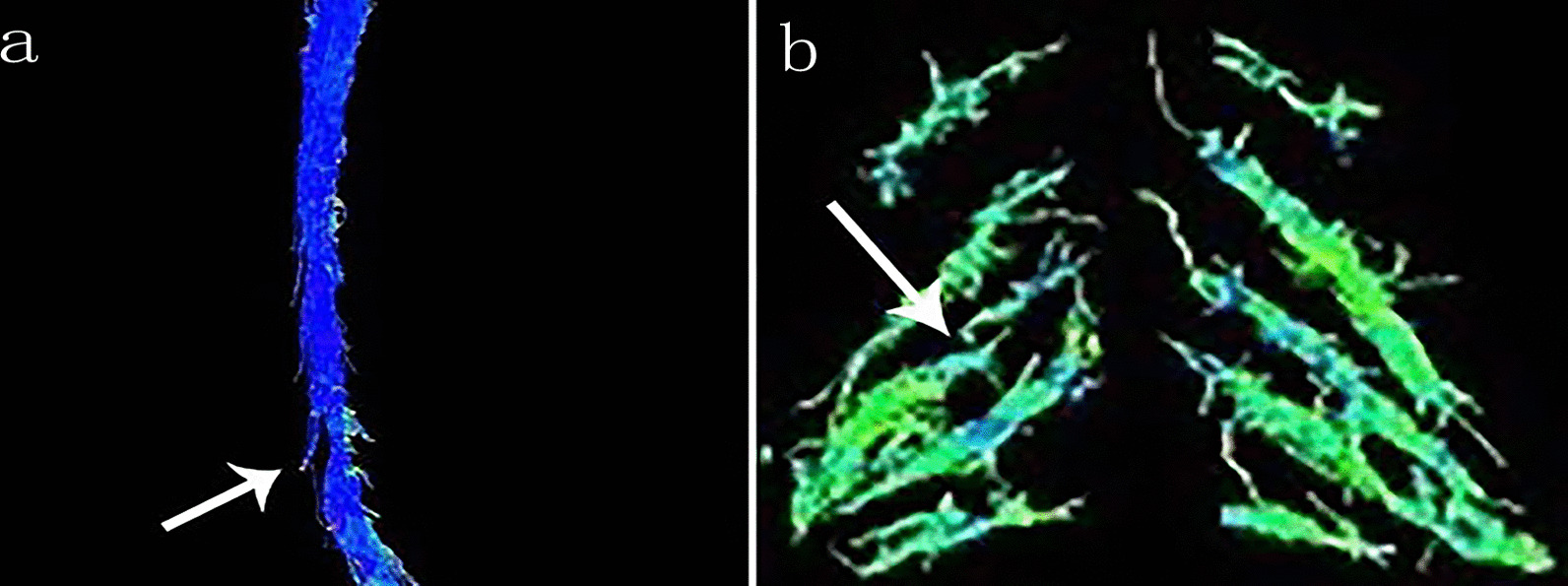
Diffusion tensor imaging in two patients with cervical spondylosis. **a** Image showing spinal cord compression at C5/6 level and spinal fiber bundle loss disorder. **b** Image showing obvious compression of the right C6 nerve root with interrupted and sparse nerve fibers

DTI tractography was successfully completed for all the participants. DTI could clearly display spinal cord and bilateral cervical nerve roots at C3/4–C6/7 level; however, C4 nerve roots were not included in the study because of their small size and difficulty to observe. In patients with CS, fibers in the compressed spinal cord and nerve roots appeared twisted, sparse, or stamped (Fig. 2).

### DTI parameters of healthy volunteers

**Fig. 3:**
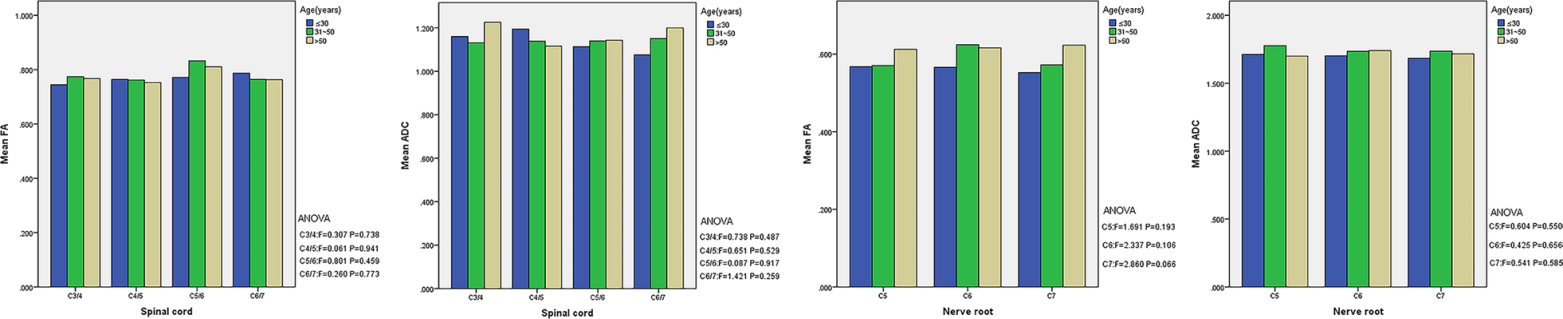
Fractional anisotropy and apparent diffusion coefficient values of the spinal cord and nerve root of healthy volunteers in different age groups (× 10–3 mm^2^/s). *P* > 0.05 is considered as statistically not significant

At different levels of cervical spinal cord and cervical nerve root, there were no significant differences in FA and ADC between different age groups among healthy volunteers (all *P* > 0.05) (Fig. 3). There were no significant differences in the FA and ADC values between the left and right nerve roots at the same level in healthy volunteers (all *P* > 0.05, Table 1).

**Table 1:** FA and ADC values of C5–C7 nerve roots in healthy volunteers (× 10^−3^ mm^2^/s)

Nerve root	FA value		*t*	*P*	ADC value		*t*	*P*
	Left	Right			Left	Right		
C5	0.587 ± 0.097	0.580 ± 0.076	0.397	0.694	1.694 ± 0.180	1.765 ± 0.276	1.626	0.115
C6	0.602 ± 0.094	0.603 ± 0.095	0.081	0.936	1.718 ± 0.132	1.733 ± 0.148	0.543	0.591
C7	0.572 ± 0.090	0.593 ± 0.109	1.063	0.297	1.702 ± 0.114	1.722 ± 0.196	0.567	0.575

*FA* anisotropic fraction, *ADC* apparent diffusion coefficient, *t* t value of t test, *P P* value

### Comparison of the DTI parameters between patients and healthy volunteers

For the cervical spinal cord, the FA at the most compressed level was significantly lower than the value at the corresponding level in healthy volunteers. In contrast, the ADC at the most compressed level was significantly higher than the value at the corresponding level in healthy volunteers (Table 2).

**Table 2:** FA and ADC values of spinal cord at C3/C4–C6/C7 of all subjects (× 10^−3^ mm^2^/s)

Spinal cord	FA value		*t*	*P*	ADC value		*t*	*P*
	HV	CS			HV	CS		
C3/4	0.762 ± 0.097	0.637 ± 0.087	3.288	0.003	1.172 ± 0.176	1.558 ± 0.294	4.369	0.000
C4/5	0.759 ± 0.076	0.638 ± 0.097	4.603	0.000	1.149 ± 0.156	1.421 ± 0.302	3.268	0.004
C5/6	0.805 ± 0.108	0.642 ± 0.075	6.765	0.000	1.132 ± 0.168	1.431 ± 0.255	5.313	0.000
C6/7	0.771 ± 0.081	0.657 ± 0.069	4.427	0.000	1.142 ± 0.169	1.373 ± 0.183	4.032	0.000

*FA* anisotropic fraction, *ADC* apparent diffusion coefficient, *HV* volunteer, *CS* cervical spondylosis, *t* t value of t test, *P P* value

For the cervical nerve root, the FA at the compressed level was significantly lower than the value at the corresponding level in healthy volunteers. In contrast, the ADC at the compressed level was significantly higher than the value at the corresponding level in healthy volunteers (Table 3).

**Table 3:** FA and ADC values of C5–C7 nerve roots of all subjects (× 10^−3^ mm^2^/s)

Nerve root	FA value		*t*	*P*	ADC value		*t*	*P*
	HV	CS			HV	CS		
C5	0.583 ± 0.087	0.452 ± 0.097	4.364	0.000	1.730 ± 0.234	1.963 ± 0.211	2.961	0.004
C6	0.602 ± 0.094	0.436 ± 0.070	8.848	0.000	1.726 ± 0.139	1.906 ± 0.166	5.082	0.000
C7	0.583 ± 0.100	0.444 ± 0.042	8.893	0.000	1.712 ± 0.159	1.933 ± 0.161	5.475	0.000

*FA* anisotropic fraction, *ADC* apparent diffusion coefficient, *HV* volunteer, *CS* cervical spondylosis, *t* t value of t test, *P P* value

### Correlations of the DTI parameters and mJOA scores in patients

**Fig. 4:**
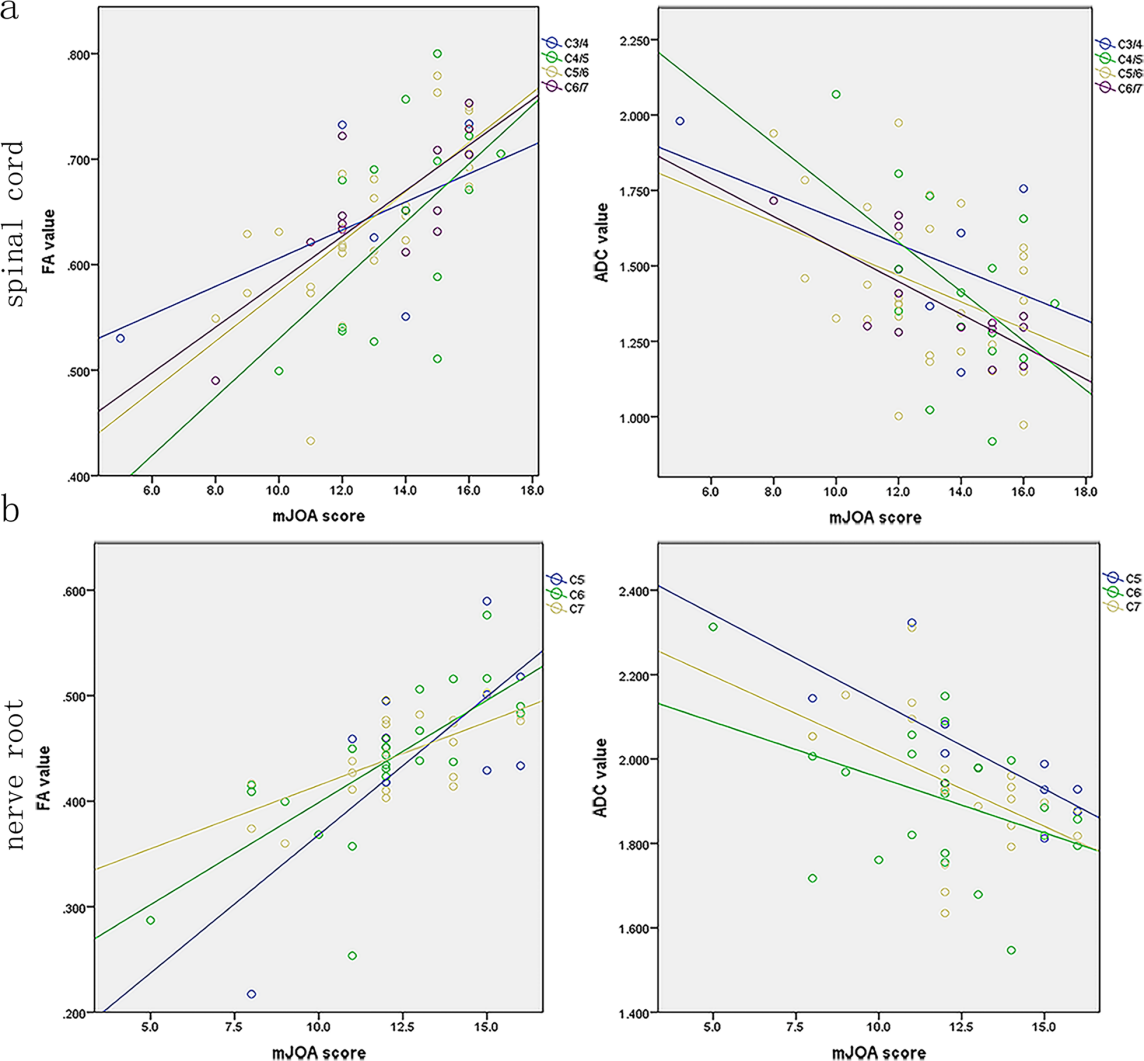
Graphical representation of the correlation analysis of the fractional anisotropy (FA) value with apparent diffusion coefficient (ADC) value and modified Japanese Orthopedic association (mJOA) score showing that the FA value was positively correlated with the mJOA score, while the ADC value was negatively correlated with the mJOA score. **a** Correlation analysis of the FA value, ADC value, and mJOA score in the spinal cord under compression. **b** Correlation analysis of the FA value, ADC value, and mJOA score in the nerve root under compression

The correlations between neurological severity and DTI metrics are shown in Fig. 4. FA and ADC were significantly correlated with mJOA score except at C3/4 level of cervical spinal cord (*P* < 0.05, respectively). Moderately positive correlation was found between FA at the most compressed level and mJOA score. By contrast, moderately negative correlation was found between ADC at the most compressed level and mJOA score (Fig. 4). FA value-mJOA score: r = 0.589, *P* = 0.219 (C3/4); r = 0.545, *P* = 0.036 (C4/5), r = 0.729, *P* = 0.000 (C5/6), r = 0.767, *P* = 0.002 (C6/7); ADC value-mJOA score: r = − 0.547, *P* = 0.261 (C3/4), r = − 0.517, *P* = 0.049 (C4/5), r = − 0.400, *P* = 0.032 (C5/6), r = − 0.719, *P* = 0.006 (C6/7); FA value-mJOA score: r = 0.706, *P* = 0.023 (C5), r = 0.722, *P* = 0.000 (C6), r = 0.679, *P* = 0.001 (C7). ADC value-mJOA score: r = − 0.731, *P* = 0.016 (C5), r = − 0.415, *P* = 0.044 (C6), r = − 0.500, *P* = 0.021 (C7). The FA value, ADC value, and mJOA score in the C3/4 segment of the spinal cord in patients with CS were not correlated with the mJOA score, possibly due to the small sample size.

### ROC curve analysis of the DTI parameters in patients

ROC analyses showed that FA values and ADC values had a good discriminatory power to differentiate healthy volunteers from patients with mild, moderate, and severe (Tables 4, 5, 6). Since the sample size of C3/4 of the spinal cord with mild compression and C3/4 and C4/5 of the spinal cord with severe compression were all 1, ROC curve analysis was not performed.

**Table 4:** Results of the ROC curve analysis in distinguishing mild cervical spondylosis

	FA value			ADC value		
	AUC	Cut off	Sensitivity/specificity	AUC	Cut off	Sensitivity/specificity
C4/5	0.786	0.722	0.857/0.667	0.743	1.186	0.857/0.633
C5/6	0.750	0.764	0.875/0.700	0.750	1.148	0.875/0.600
C6/7	0.794	0.754	1.000/0.700	0.772	1.151	1.000/0.500
C5	0.813	0.519	0.800/0.783	0.783	1.811	1.000/0.650
C6	0.812	0.518	0.750/0.800	0.821	1.794	1.000/0.733
C7	0.839	0.505	1.000/0.800	0.850	1.814	1.000/0.783

*FA* anisotropic fraction, *ADC* apparent diffusion coefficient, *AUC* area under the ROC curve

**Table 5:** Results of the ROC curve analysis in distinguishing moderate cervical spondylosis

	FA value			ADC value		
	AUC	Cut off	Sensitivity/specificity	AUC	Cut off	Sensitivity–specificity
C3/4	0.858	0.653	0.750/0.900	0.817	1.316	0.750/0.900
C4/5	0.881	0.691	0.857/0.900	0.829	1.290	0.857/0.867
C5/6	0.912	0.698	1.000/0.867	0.838	1.327	0.714/0.900
C6/7	0.793	0.649	0.800/0.933	0.933	1.273	1.000/0.833
C5	0.928	0.498	1.000/0.867	0.906	1.937	1.000/0.817
C6	0.963	0.508	1.000/0.883	0.786	1.911	0.667/0.950
C7	0.897	0.497	1.000/0.817	0.757	1.841	0.667/0.817

*FA* anisotropic fraction, *ADC* apparent diffusion coefficient, *AUC* area under the ROC curve

**Table 6:** Results of the ROC curve analysis in distinguishing severe cervical spondylosis

	FA value			ADC value		
	AUC	Cut off	Sensitivity/specificity	AUC	Cut off	Sensitivity/specificity
C5/6	0.981	0.636	1.000/0.933	0.967	1.314	1.000/0.867
C6/7	0.967	0.636	1.000/0.933	0.950	1.298	1.000/0.900
C5	0.967	0.463	1.000/0.933	0.975	2.093	1.000/0.967
C6	0.992	0.450	1.000/0.967	0.848	1.963	0.625/0.967
C7	0.956	0.441	1.000/0.917	1.000	2.046	1.000/1.000

*FA* anisotropic fraction, *ADC* apparent diffusion coefficient, *AUC* area under the ROC curve

For mild CS, the AUC of FA and ADC values to predict the severity of CS was 0.750–0.839 and 0.743–0.850, respectively; for moderate CS, the AUC of FA and ADC values to predict the severity of CS was 0.793–0.963 and 0.757–0.933, respectively; for severe CS, the AUC of FA and ADC values to predict the severity of CS was 0.956–0.992 and 0.848–1.000, respectively. The results show that FA and ADC values can predict the occurrence and development of CS well.

## Discussion

DTI is a unique method for the quantitative evaluation of nerve fiber bundles. It utilizes the anisotropy principle on the diffusion of water molecules in tissues to detect subtle structural changes in living tissues [15–17]. Recent studies have found that the FA and ADC values of DTI imaging can quantitatively evaluate the microscopic changes of spinal cord compression in cervical spondylotic myelopathy [18]. However, as the cervical nerve roots are not as bulky as the lumbosacral nerve roots, there are a few studies on cervical spondylotic radiculopathy; in most patients with cervical spondylotic myelopathy the spinal cord and nerve root are simultaneously affected. Therefore, the present study did not conduct a detailed classification of CS.

We studied the microstructural changes of the compressed spinal cord and nerve roots in CS using DTI technology, and quantified the severity of CS based on FA and ADC values. We found that compared with the corresponding segments of healthy volunteers, FA values of spinal cord and nerve root compression were lower in patients with CS, while ADC values were higher than those of healthy volunteers. It is consistent with the results of Chen et al. and Liang et al. [19, 20]. This may have resulted from a series of events; the compression of the spinal cord and nerve root leads to the blood-spinal barrier and blood-nerve barrier damage causing increased vascular permeability, which in turn causes pressure tissue edema, glial cell proliferation, decreased number and diameter of axons, and thinning of the myelin sheath. The pathological changes hinder the diffusion of water, resulting in a decrease in the FA value and increase in the ADC value [21, 22]. These results suggest that DTI parameters can quantitatively evaluate the chronic injury caused by the compression of the spinal cord and nerve root.

In our study, the average FA of the control group at different levels of the spinal cord was 0.762–0.805, the average ADC was 1.133–1.171 × 10^–3^ mm^2^/s, and the average FA of nerve roots at different levels was 0.591–0.604, the average value of ADC was 1.655–1.792 × 10^–3^ mm^2^/s. This is different from results of previous studies [23, 24], and may have resulted from the field intensity, setting of b value during DTI procedure, the selection of diffusion gradient direction, ROI selection, and other influential factors. In the study by Kerkovsky et al. [25], on the correlation between DTI and clinical manifestations, the application of the ratio of the DTI parameter, i.e., the ratio of FA and ADC values obtained at unaffected segments (C2/C3 levels) to those obtained at spinal cord compression levels, was proposed to help avoid individual variations during analysis. In addition, cervical nerve roots are much smaller than lumbosacral nerve roots and are more susceptible to cerebrospinal fluid and vascular pulsation. In our study, ROI was at the posterior ganglia of nerve roots, which reduced the influence of cerebrospinal fluid and vascular pulsation on the measurement results.

In a previous report, Sun et al. discussed surgical options for patients with radiculopathy and/or myelopathy [26], and confirmed that surgery is the best treatment for CS to reduce the risk of lifelong disability and progressive myelopathy [27]. As the outcomes of surgery are better in mild patients than in severe patients [28], evaluation of the severity of CS is of great clinical significance during surgical planning. Many studies have confirmed that DTI parameters are strongly correlated with clinical severity of patients with CS [29–32]. Jiang et al. [30] conducted a correlation study on preoperative and postoperative MRI examination, clinical evaluation, and functional recovery evaluation of 57 patients with cervical spondylotic myelopathy. The results showed that FA value was highly related to mJOA score, and FA value was better than T2 high signal intensity and sagittal canal stenosis in evaluating postoperative functional recovery. In this study, the correlation analysis of nerve function in cervical spondylotic myelopathy and/or radiculopathy with different severity was conducted and it was found that FA values decreased with the degree of nerve damage; specifically, increase in ADC values was inversely proportional with the degree of nerve damage, and ROC curve of the severity of CS was used to obtain the best cutoff value of FA and ADC. However, the findings need validated by clinical studies with large sample sizes.

Different from previous studies [18, 24], RESOLVE-DTI sequence was used in this study. RESOLVE sequence is an innovative design for K-space readout. K-space is segmented in the readout direction, Readout Partial Fourier technique is used to reduce echo interval and reduce susceptibility artifacts, and nonlinear phase correction is performed by navigation echo acquisition to reduce blur effect and improve resolution [33]. However, the scanning time of DTI sequence is longer than that of traditional single excitation.

This study had a few limitations. First, the sample size was small. More patients should be included in future studies. Second, the scanning time and post-processing time of DTI are long; the scanning parameters need to be further optimized. Partial volume effect must be avoided in ROI selection when measuring DTI parameters, because the inclusion of adjacent highly isotropic cerebrospinal fluid in ROI will significantly reduce FA and increase ADC. Therefore, selecting a smaller ROI at the lesion site is the best solution for this problem. In addition, the ROI selection of this experiment is still inadequate, that is, we only conducted segmental analysis when studying spinal cord compression. Some studies have shown that selecting appropriate ROI sets at the same segment can better describe spinal cord injury[34]. Last, most of the patients in our study had multilevel CS; we only evaluated the spinal cord segments and nerve roots with the most severe compression [35–37]. Future studies should consider the multiple levels of CS separately. Although DTI quantitative analysis can objectively evaluate the severity of CS, there is no authoritative quantitative analysis model at present, which limits its wide application in clinical work, and requires operators to have a full understanding of DTI acquisition conditions and anatomical basis of CS. It is believed that with the increase of studies and the establishment of models, DTI parameters for quantitative evaluation of the severity of CS will be widely used in clinical practice.

## Conclusion

In summary, DTI can noninvasively evaluate the microstructural changes in compressed spinal cord and nerve roots in CS of varying severity. DTI technology can intuitively display the morphology of the spinal cord and nerve root fiber bundles from multiple angles, which is a potential tool for evaluating the severity of CS.

## Data Availability

All data generated or analyzed during this study are included in this article.

## Abbreviations

ADC: Apparent diffusion coefficient
AUC: Area under curve
CS: Cervical spondylosis
DTI: Diffusion tensor imaging
DTT: Diffusion tensor tractography
FA: Fractional anisotropy
FOV: Field of view
HV: Healthy volunteer
MRI: Magnetic resonance imaging
mJOA: Modified Japanese Orthopaedic Association scores
ROC: Receiver operator characteristic
ROI: Region of interest
ET: Echo time
RT: Repetition time
TSE: Turbo spin echo
T1WI: T1 weighted imaging
T2WI: T2 weighted imaging
